# Prenatal diagnosis of fetal growth restriction with polyhydramnios, etiology and impact on postnatal outcome

**DOI:** 10.1038/s41598-021-04371-9

**Published:** 2022-01-10

**Authors:** Adeline Walter, Elina Calite, Christoph Berg, Ulrich Gembruch, Andreas Müller, Annegret Geipel

**Affiliations:** 1 Department of Obstetrics and Prenatal Medicine, University Hospital Bonn, Venusberg-Campus 1, 53127 Bonn, Germany; 2 Department of Neonatology and Pediatric Intensive Care Medicine, University Hospital Bonn, Venusberg-Campus 1, 53127 Bonn, Germany; 3 Department of Obstetrics and Prenatal Medicine, University Hospital Cologne, Kerpener Straße 34, 50931 Cologne, Germany

**Keywords:** Diseases, Medical research, Risk factors, Signs and symptoms

## Abstract

To assess the spectrum of different etiologies, the intrauterine course, outcome and possible prognostic markers in prenatally detected fetal growth restriction (FGR) combined with polyhydramnios. Retrospective study of 153 cases with FGR combined with Polyhydramnios diagnosed by prenatal ultrasound over a period of 17 years. Charts were reviewed for ultrasound findings, prenatal and postnatal outcome. All cases were categorized into etiological groups and examined for differences. Five etiological groups were identified: chromosomal anomalies (n = 64, 41.8%), complex malformation syndromes (n = 37, 24.1%), isolated malformations (n = 24, 15.7%), musculoskeletal disorders (n = 14, 9.2%) and prenatal non-anomalous fetuses (n = 14, 9.2%). Subgroups showed significant disparities in initial diagnosis of combination of both pathologies, Ratio AFI/ gestational weeks and Doppler ultrasound examinations. Overall mortality rate was 64.7%. Fetuses prenatally assigned to be non-anomalous, showed further complications in 42.9% (n = 6). Fetuses prenatally diagnosed with FGR combined with polyhydramnios are affected by a high morbidity and mortality. Five etiologic groups can be differentiated, showing significant disparities in prenatal and postnatal outcome. Even without recognizable patterns prenatally, long-term-follow up is necessary, as neurodevelopmental or growth delay may occur.

## Introduction

Fetal growth restriction (FGR) is described with an incidence of 5–10% leading to a significant risk of perinatal mortality, neonatal morbidity and long-term health defects^1–3^. The most common cause of FGR is placental insufficiency, resulting in fetal hypoxemia^2,4^. Consequently, redistribution in fetal circulation with an impaired fetal renal perfusion occurs^5^. Hence a reduced amniotic fluid up to oligohydramnios is typically observed in cases with FGR^6^.

Increased volume of amniotic fluid combined with FGR is therefore unusual and prenatally rarely seen. The reported prevalence ranges from 4 to 6% in fetuses affected by an intrauterine growth restriction^7,8^.

Considering that polyhydramnios describes an independent risk factor for an adverse perinatal outcome, a combination of both conditions seems to have a more serious negative impact on fetal outcome^8–14^. A higher rate of congenital malformations, chromosomal abnormalities, even if undetectable by ultrasound, is frequently encountered. Further maternal complications as a higher rate of cesarean sections, premature rupture of membrane, premature delivery and postpartum bleeding are reported^8,9,15^.

In the current literature, either the spectrum of a FGR or the spectrum of a polyhydramnios is predominantly described. Previously published data evaluating the combination of both pathologies is limited and is focusing more on the impact on perinatal outcome than on prenatal counselling^8,9^. Investigating fetal diagnosis leading to this unusual combination remains therefore still challenging.

In order to provide data on a large cohort with prenatally diagnosed FGR combined with polyhydramnios, we conducted a retrospective study at our tertiary referral center.

We aimed to assess the spectrum of the different etiologies, to evaluate possible prognostic parameters and postnatal outcomes of affected fetuses. In order to improve prenatal counselling, all cases were assigned into various etiological groups and examined for significant disparities.

## Materials and methods

We conducted a single-center retrospective analysis of prospective collected data between 2003 and 2019. Patients were only included in the study if they had a singleton pregnancy of certain gestational age and if prenatal sonographic examination revealed the diagnosis of FGR combined with polyhydramnios. Diagnosis had to be persistent for the rest of the pregnancy and confirmed by at least two examinations. Cases were excluded from the study, if multiple gestation was present (n = 53), combination of both pathologies were only detected in one scan (n = 17), if fetuses had unreliable measurements due to abdominal wall defects, such as omphalocele or gastroschisis (n = 2) and if postpartum outcome could not be evaluated (n = 2).

FGR was defined as an estimated fetal weight (EFW) at or below the 3rd percentile compared to normal fetal weight for gestational age, or as fetuses with of an EFW at or below the 10th percentile in combination with an abnormal Doppler or having a growth restriction in subsequent scans crossing centiles by more than two quartiles.

Polyhydramnios was defined as an amniotic fluid index (AFI) > 95th percentile of the appropriate reference range for gestational age or as a vertical deepest pocket measuring at least 8 cm^16,17^. A ratio of AFI divided by the number of gestational weeks (cm/week) was calculated for each patient.

Data were extracted from ultrasound reports in our database by Viewpoint (v. 5.0, GE Healthcare, Chicago, IL, US char). First scan showing fetal growth restriction combined with polyhydramnios was used for data acquisition on prenatal outcome. Fetal and maternal Doppler parameters were analyzed. Mean value of both uterine arteries was determined for each patient. As Doppler indices change with gestational age, values were converted into z-scores. Fetal weight was estimated using the method of Hadlock et al. and afterwards transformed into percentiles to correct for gestational age^18,19^.

Cases were categorized into five etiological groups (EG) depending on prenatal sonographic findings, genetic results if available, or neonatal outcome: chromosomal abnormalities (CA), complex malformation syndromes, including associations and anomalies of more than two organ systems (S), isolated malformations (IM), musculoskeletal disorders (MSD) and parentally non-anomalous fetuses (H).

All diagnoses made pre- and postnatal of all live-born children were compared to assess the accuracy of prenatal diagnosis. Further pregnancy outcome was classified into four groups: termination of pregnancy (TOP), intrauterine fetal (IUFD) or neonatal death (NND) and survivors. Neonatal death was defined as death within the first 28 days of life. Information on the neonatal morbidity of the non-anomalous group was requested by pediatric reports.

All methods were carried out in accordance with relevant guidelines and regulations. Experimental protocols were approved by Department of Prenatal Medicine and Obstetrics at the University of Bonn, not including an Ethical consent, as the Ethics Committee of the University of Bonn does not request formal approval for an anonymized retrospective analysis of clinical data.

Statistical analysis was performed using SPSS (v23.0, IBM, Armonk, NY, US). Outcomes were quantified as means with standard deviation (SD) for continuous variables, median with range, and percentages for categorical variables. Fisher’s exact test and Chi-squared (χ^2^) test was applied to verify the association between the categorical variables and the EG. One-way ANOVA with post hoc test was applied to verify the differences among the different etiologies. P < 0.05 was considered significant.

### Statement of approval

IRB of the Department of Prenatal Medicine and Obstetrics at the University Hospital of Bonn approved the Experimental protocols, without an Ethical consent, as the Ethics Committee of the University of Bonn does not request formal approval for an anonymized retrospective analysis of clinical data.

### Statement of ethics

As the Ethics Committee of the University of Bonn does not request formal approval for an anonymized retrospective analysis of clinical data, Ethical consent was not required.

### Consent to participate

Informed consent was obtained from every patient participating in this Study for clinical data collection, analysis and the use of those data for research.

### Consent for publication

Consent for publication was obtained from every patient participating in this Study.

## Results

During the period of 17 years, 4898 patients with prenatally diagnosed FGR were consulted at the University Hospital Bonn. With an incidence of 4.6%, a total of 227 cases were diagnosed prenatally combined with polyhydramnios. According to inclusion criteria a total of 153 cases were recruited into the study.

The following groups of different etiologies were identified: CA (n = 64, 41.8%), S (n = 37, 24.1%), IM (n = 24, 15.7%), MSD (n = 14, 9.2%) and H (n = 14, 9.2%) (Table 1). Numerical chromosomal anomalies represented the largest number within the CA group (95.3% numerical vs. 4.7% structural chromosomal anomalies).

**Table 1:** Distribution of the different etiologies groups (EG).

EG	Findings		n (%)
**CA**			64 (41.8)
	Trisomy 18		47 (73.4)
	Trisomy 21		3 (4.7)
	Trisomy 14, 9, 2		Each 1 (1.6)
	Klinefelter syndrome		1 (1.6)
	Triploidy		1 (1.6)
	Trisomy mosaicism (9, 16, Turner syndrome (45, X0/46, XX))		3 (4.7)
	Partial monosomy (monosomy 15q (n = 2), monosomy 14q, del. Chr. 13, 18 (n = 1, each))		5 (7.8)
	Structural chromosomal aberration (trans. Chr. 17)		1 (1.6)
**S**			37 (24.1)
	VACTERL		7 (18.9)
	Cornelia de Lange syndrome		4 (10.8)
	Miller-Dieker syndrome		4 (10.8)
	Kabuki-, Rubinstein-Taybi-, Cantrell		Each 1 (2.7)
	Other		19 (51.4)
**IM**			24 (15.7)
	Congenital diaphragmatic hernia (CDH)		10 (41.7)
	Gastrointestinal malformations	Duodenal atresia (2) Esophageal atresia (4)	6 (25.0)
	Congenital heart disease		7 (29.2)
	Other	Cervical lymphangioma (1)	1 (4.2)
**MSD**			14 (9.2)
	Fetal akinesia deformation sequence (FADS)		9 (64.3)
	Skeletal disorders (SD)		5 (35.7)
		Achondroplasia (1) Jeune- (1) Others, non-lethal type (3)	
**H**			14 (9.2)

Maternal characteristics showed no significant differences among the etiological groups.

**Figure 1:**
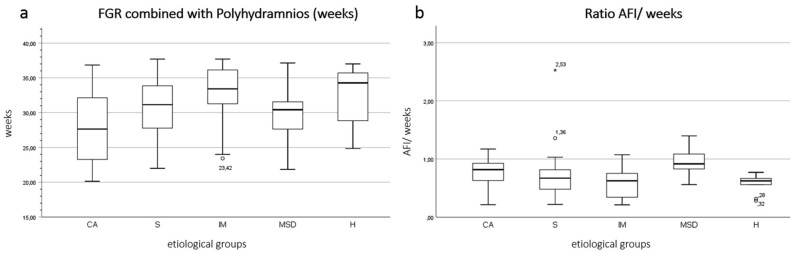
In **(a,b)**, boxplots display differences between the five etiological groups. FGR combined with polyhydramnios was earlier detected in the CA group (27.8 weeks) and later in the H (32.4 weeks) and IM group (32.6 weeks). Subgroup comparison was significant (p = 0.01) **(a)**. MSD group showed a significant higher ratio AFI/ gestational week (0.9) than the other groups (p = 0.02) **(b)**.

Prenatal characteristics of the different disease entities are presented in Table 2. Initial diagnosis of FGR and the common occurrence with polyhydramnios varied significantly among the groups (p = 0.03 and p = 0.01) (Fig. 1a). The calculated ratio (AFI/gestational weeks) also deviated significantly in the subgroups (p = 0.02) (Fig. 1b). The MSD-group revealed the highest and the H group the lowest ratio (0.9 vs. 0.6). Doppler examination showed further considerable differences in the z-scores of the pulsatility Index (PI) of the uterine artery (Ut) (p = 0.02). PI-Ut z-score was significantly increased in the H group (2.1 ± 1.7).

**Table 2:** Prenatal characteristics of the different disease entities.

Mean ± SD						p	Post-hoc	p
Parameter	CA	S	IM	MSD	H			
FGR diagnosis (weeks)	27.2 (± 4.6)	27.6 (± 4.3)	30.4 (± 4.5)	28.2 (± 3.5)	29.9 (± 5.7)	**0.03**	CA vs. IM CA vs. H	**0.02** **0.04**
FGR with polyhydramnios (weeks)	27.8 (± 4.8)	30.8 (± 3.8)	32.6 (± 4.5)	30.2 (± 4.5)	32.4 (± 4.3)	**0.01**	CA vs. IM CA vs. H	**0.04** **0.04**
AFI (cm)	23.1 (± 6.2)	25.4 (± 8.9)	24.7 (± 4.7)	29.3 (± 9.2)	22.4 (± 3.2)	**NS**		
Ratio (AFI/gestational week)	0.7 (± 0.3)	0.7 (± 0.4)	0.6 (± 0.2)	0.9 (± 0.2)	0.6 (± 0.2)	**0.02**	MSD vs. IM MSD vs. H	**0.04** **0.01**
Ut PI Z-score	0.9 (± 1.6)	0.8 (± 1.2)	1.0 (± 1.2)	0.6 (± 1.3)	2.1 (± 1.7)	**0.02**	MSD vs. H	**0.03**
UA PI Z-score	0.9 (± 1.9)	1.0 (± 1.6)	1.7 (± 1.8)	0.8 (± 1.5)	1.9 (± 1.9)	NS		
Birth weight Perc	4.5 (± 2.8)	4.6 (± 3.0)	5.8 (± 3.3)	7.6 (± 8.3)	4.9 (± 2.9)	NS		

Significant values are in bold.

*CA* chromosomal anomalies, *S* complex malformation syndromes, *IM* isolated malformation, *MSD* musculoskeletal disorders, *H* prenatal non-anomalous fetuses, *FGR* fetal growth restriction, *AFI* amniotic fluid index, *Ut PI* pulsatility index of uterine arteries, *UA PI* pulsatility index of umbilical artery, *SD* standard deviation.

Invasive testing varied substantially within the subgroups (p < 0.001). Overall genetic testing was conducted in 77.8% (n = 119) although recommended in all cases. No further clarification was carried out in 22.2% (n = 34), because of a missing consequence for the parents.

In all other cases conventional karyotyping was performed, leading to a diagnosis in 86.6% (n = 103) cases. Further genetic testing was based on abnormal sonographic findings.

Chromosomal microarray (CMA) was carried out in 16% (n = 19). Next-generation sequencing (NGS) using different panels, was applied in 5.9% (n = 7) of cases. In 9.2% (n = 11) chromosomal aberration was only detected by CMA and in 3.4% (n = 4) by NGS. In three cases the final genetic diagnosis was made postnatal. Prenatal genetic testing was unremarkable, but postnatal course conspicuous. Further genetic tests lead to the diagnosis of Cantrell Core Myopathia, Rubin-Taybi syndrome, Cornelia de Lange syndrome. The H group revealed the smallest proportion of invasive testing (2/14; 14.3%). In both cases prenatally a severe FGR was suspected (< 3rd percentile). In one case, the fetus showed an increased nuchal translucency (NT). A chorionic villous sampling (CVS) was obtained at 11 weeks of gestation, with an inconspicuous result. In the further course pathological Doppler velocimetry and fetal bradycardia occurred so delivery took place in the 26th week. Postnatal course of the extremely premature infant showed typical complications of preterm birth (Intraventricular hemorrhage (IVH), necrotizing enterocolitis (NEC)), with an otherwise unremarkable current condition. In the other case, amniocentesis (AC) was performed in week 33, showing also an uneventful result. Due to an abnormal Doppler, the child was born in week 36. Speech developmental disorder and unclear epilepsy was diagnosed within the first year of life.

**Figure 2:**
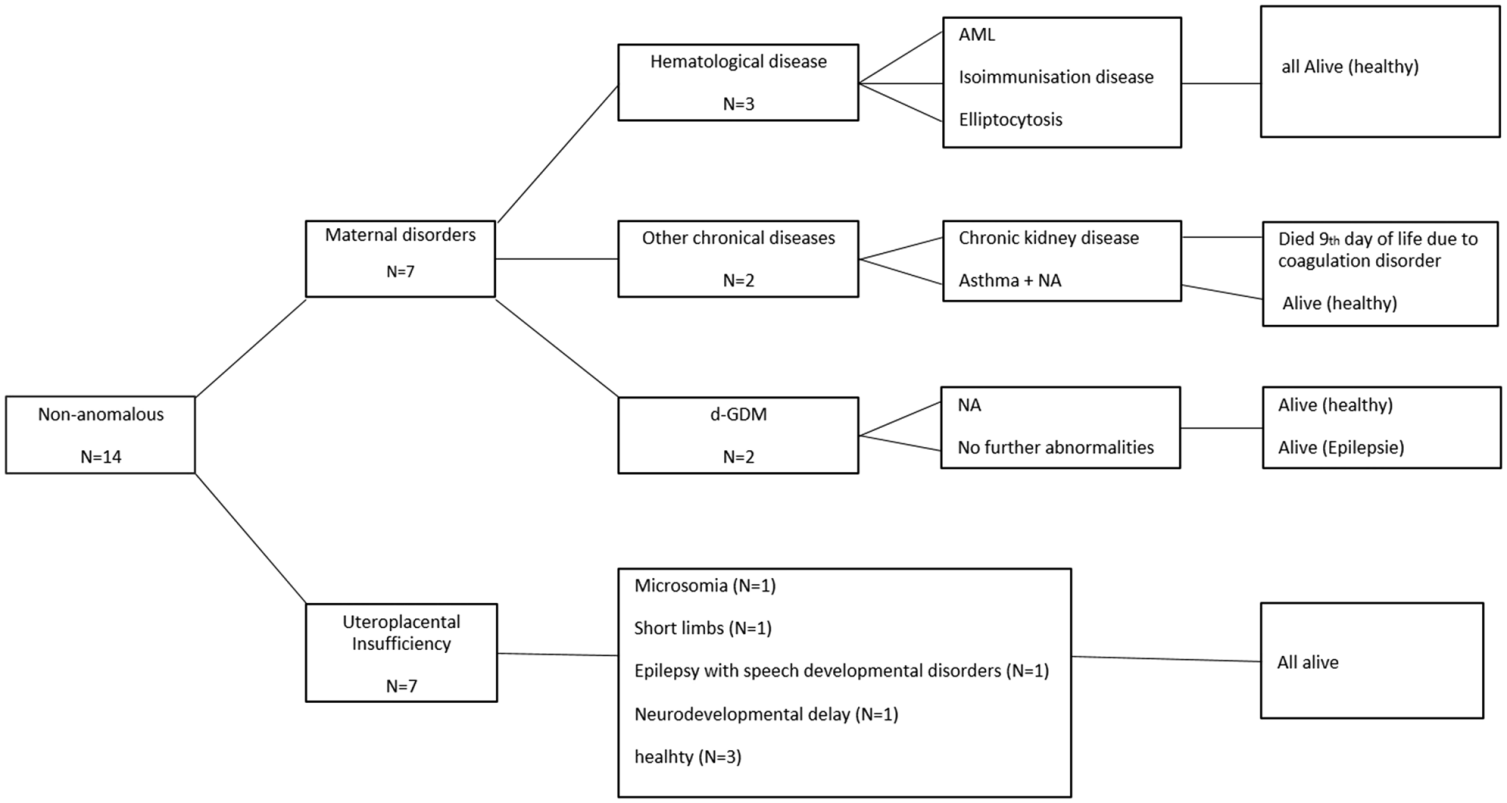
Flowchart demonstrating details on outcome and postnatal course of the non-anomalous fetuses. *NA* nicotine abuse, *d-GDM* dietetic-dependent gestational diabetes (diagnosed with a 75-g oral glucose tolerance test (oGTT), (thresholds ≥ 92/180/155 mg/dl) according to the German maternity guidelines^25^.

Table 3 outlines postnatal outcome of the different groups, representing an overall mortality rate of 64.7%. Figure 2 shows details on the postnatal course of the H group. In only three cases, where maternal influencing factors were excluded, follow up was unremarkable. Influencing factors were either hematological or based on maternal chronic diseases.

**Table 3:** Associated condition and postnatal outcome of the different disease entities.

Etiological group	Total	TOP	IUFD	NND	Alive till discharge
CA	64	67.2% (43/64)	9.4% (6/64)	14.1% (9/64)	9.4% (6/64)
S	37	29.7% (11/37)	2.7% (1/37)	29.7% (11/37)	37.9% (14/37)
IM	24	–	–	25.0% (6/24)	75.0% (18/24)
MSD	14	57.2% (8/14)	–	21,4% (3/14)	21.4% (3/14)
H	14	–	–	7.1% (1/14)	93.9% (13/14)

*IUFD* intrauterine fetal death, *NND* neonatal death, *TOP* termination of pregnancy, *CA* chromosomal anomalies, *S* complex malformation syndromes, *IM* isolated malformation, *MSD* musculoskeletal disorders, *H* prenatal non-anomalous fetuses.

## Discussion

According to the literature, FGR combined with a polyhydramnios complicates 0.2–6.0% of pregnancies^7–9,15^. In our study we calculated an incidence of 4.6%. Initial prenatal detection of FGR combined with polyhydramnios was 29.7 weeks. Compared to previously published studies initial detection of both pathologies was 32.7 weeks^8^. As these studies were conducted before 2000, poorer ultrasound technologies and the changed approach of consultation in prenatal tertiary centers might explain the differences^8,9^.

Chromosomal anomalies represented the highest proportion in our study population (41.8%). Trisomy 18 was found in 73.4% of fetuses assigned to the CA group, whereas structural chromosomal aberration was seen in 1.6%. These results are consistent with the analysis of Sickler et al. and Snijders et al., both reporting nearly 40% showing chromosomal anomalies^8,15^. Trisomy 18 was also the most frequently detected numerical chromosomal aberration in their studies (66.7%, 47.1%), whereas structural aberration were rarely seen^8^.

Further Sickler et al. reported 92% (n = 36) of the examined fetuses showing further anomalies, including skeletal dysplasia in six cases and arthrogryposis in one case^8^. Comparable to our results, we also observed further anomalies in 90.9%. In relation to the recorded anomalies, Sickler et al. described 24 out of 38 cases to have cardiac abnormalities (63.2%). Differently, congenital diaphragmatic hernia (CDH) were the most common isolated malformations with 41.7% (10/24) in our study. Cardiac malformations were represented with 29.2% (7/24). The difference in results may be explained by the analysis of the entire study collective by Sickler et al. including also fetuses with chromosomal aberrations, as trisomy 18^8^. Regarding perinatal mortality rate Sickler et al. estimated it to be 59%^8^. Whereas other published data, such as Furman et al. found the mortality rate to be 9.6%, after exclusion of congenital malformations^9^. These results correspond to our data with an overall mortality rate of 64.7% and 7.1% after eliminating congenital malformation.

In accordance with the American College of Obstetricians and Gynecologists (ACOG) and the Royal College of Obstetricians and Gynecologists (RCOG), prenatal genetic diagnostic testing was recommended in all our cases, when the combination of both pathologies was observed in the midtrimester or further structural anomalies were present^2,20^. We found a 9.2% and 3.4% incremental yield by CMA and NGS compared to conventional karyotype. This is consistent with the result of Borrell et al., but lower compared to the findings of Hay et al., who described an incremental yield by CMA of 16%^21–23^. These differences might be explained by several reasons: (1) different inclusion criteria of the study populations; (2) CMA is currently not a coverage of the public health insurance companies in Germany and therefore not routinely performed; (3) not all patients with an unremarkable karyotype had received a CMA. Estimation of the effective additional benefit remains difficult and might be higher. Nevertheless, our findings, indicates that CMA/ NGS should be recommended, especially in cases with FGR combined with polyhydramnios and normal karyotypes, as they might provide incremental yield (up to nearly 13.4%) of detecting chromosomal abnormalities and change parental counselling.

In our study we were able to demonstrate that prenatal detection of FGR combined with polyhydramnios should lead the investigator to think of different etiological groups. Fetuses assigned to the CA group were identified significantly earlier (p = 0.01) compared to all the other EGs. In addition, we found that an extremely increased AFI combined with a mild FGR (8.0 ± 8.6) and unremarkable Doppler examination should lead the investigator to think of a possible musculoskeletal disorder. In non-anomalous fetuses, FGR and polyhydramnios were detected later (33 weeks) and of a milder severity compared to the other subgroups (p = 0.03). Further, we found significant increased Doppler values of the uterine artery (p = 0.04). Affected patients were predominantly primigravida, a preeclampsia occurred in 7.1%. In 50.0% maternal diseases were able to explain the occurrence of this rare combination. In the remaining 50.0% fetal neurological impairments were found in 14.3%, endocrinological impairments in 7.1% and in 7.1% skeletal abnormality was suspected, although it is uncertain whether they should be better categorized in the MSD group instead.

The strength of our current study is that it represents one of the largest cohort of prenatally diagnosed FGR combined with polyhydramnios and the first evaluation of the spectrum of the different etiologies and examination of them for significant disparities. Further, first time follow up of the survivors was evaluated over a period from 1 to 14 years (mean 5.8 years).

Nevertheless, our study has certain limitations. First, it is a retrospective analysis. Second, the outcome of fetuses assigned to the H group, was requested in a not standardized procedure, so neurodevelopmental follow up was not clearly defined. Third, although measurement of AFI is superior for identification of polyhydramnios and was used for our analysis, it might be influenced by the different investigators and thus our results of the subgroup comparisons^24^.

In conclusion prenatal detection of an FGR combined with polyhydramnios represents a rare phenomenon with an extremely high mortality rate. If non-anomalous fetuses are observed, maternal history should be investigated especially for haematological diseases. An early referral to a perinatal tertiary center should be performed and an invasive testing should be recommended. Further genetic testing (CMA/NGS) should be performed, especially if the karyotype is unremarkable. Detailed prenatal neurosonography might be helpful. Based on our data, these patients should receive long-term follow up, with emphasis on endocrinological and neurodevelopmental assessment. Signs of neurodevelopmental or growth delay should be recognized. As these disorders may affect quality of live, early detection is crucial. Nevertheless, further prospective studies are necessary.

## Data Availability

The datasets generated during and/or analyzed during the current study are available from the corresponding author on reasonable request.

## References

[1] Albu AR, Anca AF, Horhoianu VV, Horhoianu IA (2014). Predictive factors for intrauterine growth restriction. J. Med. Life.

[2] ACOG Practice Bulletin No. 204 (2019). Fetal growth restriction. Obstet. Gynecol..

[3] Crispi F, Miranda J, Gratacós E (2018). Long-term cardiovascular consequences of fetal growth restriction: Biology, clinical implications, and opportunities for prevention of adult disease. Am. J. Obstet. Gynecol..

[4] Nardozza LMM (2017). Fetal growth restriction: Current knowledge. Arch. Gynecol. Obstet..

[5] Zhong Y, Tuuli M, Odibo AO (2010). First-trimester assessment of placenta function and the prediction of preeclampsia and intrauterine growth restriction. Prenat. Diagn..

[6] Yamamoto R, Ishii K, Nakajima E, Sasahara J, Mitsuda N (2018). Ultrasonographic prediction of antepartum deterioration of growth-restricted fetuses after late preterm. J. Obstet. Gynaecol. Res..

[7] Eydoux P (1989). Chromosomal prenatal diagnosis: Study of 936 cases of intrauterine abnormalities after ultrasound assessment. Prenat. Diagn..

[8] Sickler GK, Nyberg DA, Sohaey R, Luthy DA (1997). Polyhydramnios and fetal intrauterine growth restriction: Ominous combination. J. Ultrasound Med..

[9] Furman B (2000). Hydramnios and small for gestational age: Prevalence and clinical significance. Acta Obstet. Gynecol. Scand..

[10] Luo Q-Q (2017). Idiopathic polyhydramnios at term and pregnancy outcomes: A multicenter observational study. J. Matern. Fetal. Neonatal. Med..

[11] Kornacki J, Adamczyk M, Wirstlein P, Osiński M, Wender-Ożegowska E (2017). Polyhydramnios—Frequency of congenital anomalies in relation to the value of the amniotic fluid index. Ginekol. Pol..

[12] Hamza A, Herr D, Solomayer EF, Meyberg-Solomayer G (2013). Polyhydramnios: Causes, diagnosis and therapy. Geburtshilfe Frauenheilkd.

[13] Adamczyk M, Kornacki J, Wirstlein P, Szczepanska M, Wender-Ozegowska E (2019). Follow-up of children with antenatally diagnosed idiopathic polyhydramnios. Ginekol. Pol..

[14] Lazebnik N, Many A (1999). The severity of polyhydramnios, estimated fetal weight and preterm delivery are independent risk factors for the presence of congenital malformations. Gynecol. Obstet. Invest..

[15] Snijders RJ, Sherrod C, Gosden CM, Nicolaides KH (1993). Fetal growth retardation: Associated malformations and chromosomal abnormalities. Am. J. Obstet. Gynecol..

[16] Phelan JP, Smith CV, Broussard P, Small M (1987). Amniotic fluid volume assessment with the four-quadrant technique at 36–42 weeks’ gestation. J. Reprod. Med..

[17] Chamberlain PF, Manning FA, Morrison I, Harman CR, Lange IR (1984). Ultrasound evaluation of amniotic fluid volume. II. The relationship of increased amniotic fluid volume to perinatal outcome. Am. J. Obstet. Gynecol..

[18] Hadlock FP, Harrist RB, Martinez-Poyer J (1991). In utero analysis of fetal growth: A sonographic weight standard. Radiology.

[19] Arduini D, Rizzo G (1990). Normal values of pulsatility index from fetal vessels: A cross-sectional study on 1556 healthy fetuses. J. Perinat. Med..

[20] Sagi-Dain L, Peleg A, Sagi S (2017). Risk for chromosomal aberrations in apparently isolated intrauterine growth restriction: A systematic review. Prenat. Diagn..

[21] Hay SB (2018). ACOG and SMFM guidelines for prenatal diagnosis: Is karyotyping really sufficient?. Prenat. Diagn..

[22] Borrell A, Grande M, Pauta M, Rodriguez-Revenga L, Figueras F (2018). Chromosomal microarray analysis in fetuses with growth restriction and normal karyotype: A systematic review and meta-analysis. Fetal. Diagn. Ther..

[23] Vanlieferinghen S (2014). Second trimester growth restriction and underlying fetal anomalies. Gynecol. Obstet. Fertil..

[24] Hughes DS (2020). Accuracy of the ultrasound estimate of the amniotic fluid volume (amniotic fluid index and single deepest pocket) to identify actual low, normal, and high amniotic fluid volumes as determined by quantile regression. J. Ultrasound Med..

[25] Schäfer-Graf UM (2018). Gestational diabetes mellitus (GDM)—Diagnosis, treatment and follow-up guideline of the DDG and DGGG (S3 level, AWMF registry number 057/008, February 2018). Geburtshilfe Frauenheilkd.

